# Dissipation Behavior of Three Pesticides in Prickly Pear (*Opuntia ficus-indica* (L.) Mill.) Pads in Morelos, Mexico

**DOI:** 10.3390/ijerph16162922

**Published:** 2019-08-15

**Authors:** Irene Iliana Ramírez-Bustos, Hugo Saldarriaga-Noreña, Ernesto Fernández-Herrera, Porfirio Juárez-López, Iran Alia-Tejacal, Dagoberto Guillén-Sánchez, Ismael Rivera-León, Víctor López-Martínez

**Affiliations:** 1 Posgrado en Ciencias Agropecuarias y Desarrollo Rural, Facultad de Ciencias Agropecuarias, Universidad Autónoma del Estado de Morelos (UAEM), Av. Universidad 1001, Col. Chamilpa, Cuernavaca, Morelos 62209, México; 2 Centro de Investigaciones Químicas, Instituto de Ciencias Básicas y Aplicadas (UAEM), Av. Universidad 1001, Col. Chamilpa, Cuernavaca 62209, Mexico; 3 Universidad de Sonora, Departamento de Agricultura y Ganadería, Carretera Bahía de Kino km 21. Hermosillo, Sonora 83000, México

**Keywords:** pesticide residues, dissipation, QuEChERS, food-safety

## Abstract

The dissipation of three field-applied pesticides (chlorothalonil, chlorpyrifos, and malathion), on cultivated prickly pear (*Opuntia ficus-indica* (L.) Mill.) pads was studied. The extraction of pesticides was carried out using the European quick, easy, cheap, effective, rugged, and safe (QuEChERS) extraction technique and detection was carried out using tandem liquid chromatography with mass spectrometry. At harvest, 15 days after application, pesticide dissipation was below the level of detectability. Dissipation curves for prickly pear pads fit to a first-order kinetic equation. Two initial concentration levels were used for each pesticide. The approximate dissipation time for all pesticides studied was similar (10 days) and the half-life time was around six days. Final concentrations for the three pesticides were below the reference maximum residue level (MRL) (0.01 mg/kg), which suggests that these products can be applied safely in the commercial production of prickly pear pads at the established concentrations.

## 1. Introduction

One of the cactus species with the greatest economic importance in the world is the prickly pear (*Opuntia ficus-indica* (L.) Mill.) [1]. In 2017, 12,620.4 hectares were cultivated in 26 states of Mexico, with a volume of 810,938.99 tons [2]. This crop is affected by many pests and diseases, including the cactus weevil, *Metamasius spinolae* (Gyllenhal) [3,4,5], and the mealybug *Dactylopius opuntiae* (Cockerell) [6]. Although there are no authorized pesticides in Mexico for their control, this fact does not prevent producers from using chemical products [7].

To enhance productivity and protect the crop against pests and disease, different pesticides are currently applied at different stages of the production chain. In the field, when the vegetable is still developing, pesticides from different chemical families are applied [8,9]. For this reason, there is the possibility of finding residues in the harvested crop, and human beings are exposed to these substances [10]. The dissipation rates of pesticide active substances found in crops are regulated by the combination of natural factors such as biodegradation, photodegradation, and chemical hydrolysis, which decrease its persistence [11]. These processes favor the excitation, rupture, and/or rearrangement of chemical bonds, which lead to the partial transformation of the parental compounds [12]. Only its mineralization, in which H_2_O, CO_2,_ and other minerals are produced, ensures the reduction or elimination of the toxic pesticide effects [13]. Its persistence is related to the efficiency of the transformation processes in natural conditions [14]. The primary route of malathion degradation in soil is through aerobic soil metabolism. The half-life of malathion in soil was reported to be three days in alkaline soil and seven days in acidic soil. Biodegradation by microorganisms is an important fate process in soil, mainly when pH < 7 [15]. Meanwhile, chlorpyrifos presents a great variation in its degradation. This variation has been attributed to the behavior of factors such as pH, temperature, moisture content, organic carbon content, and pesticide formulation [16,17]. Initially, the high rate of chlorpyrifos degradation in soils with alkaline pH was attributed to chemical hydrolysis. Later, it was concluded that the relationship between high soil pH and chemical hydrolysis was weak, since there was little degradation in several high-pH soils when sterile [18]. The degradation of chlorothalonil in soil is mainly due to microbial activity, and the main metabolite reported is 4-hydroxy-2,5,6-trichloroisophthalonitrile (OH-CT) [19]. For malathion, two pathways are reported—photolysis (with a posterior formation of trimethyl impurities) [20] and through soil bacteria [21]. 

The dissipation rate of pesticides in crops is expressed as the pesticide half-life (residual lifetime, RL50). The half-life is defined as the time required for the pesticide residue level to fall by half of the initial concentration after its application [22,23], which constitutes an important parameter that is used to establish the pre-harvest interval, as well as the adjustment to accomplish the legal Maximum Residue Levels (MRLs) [24]. A key issue for establishing accurate and safe pre-harvest intervals is to realize that pesticide dissipation depends not only on the chemical properties of the compound, but also on the environmental conditions that rule their behavior [24].

In Mexico, there are no authorized pesticides in prickly pear pad production, so the period between application and harvest is unknown. Considering that the amount of pesticide residue present in a vegetable after application depends on the dose, among other factors, it is important to know how these residues vary as a function of time to calculate the period of dissipation and thus determine the pre-harvest interval [25]. The aim of this study was to obtain the dissipation curves of chlorpyrifos ethyl, malathion, and chlorothalonil for the maximum and minimum doses commonly used by nopal vegetable producers in the Mexican state of Morelos.

The analytical techniques that have shown the best results for this type of compound are gas chromatography and liquid chromatography, both coupled to mass spectrometry, given their high sensitivity and selectivity. Prior to chromatographic analysis, the exhaustive purification of the extracts is required, specifically in complex matrices, with the intention of eliminating the effects of the matrix caused by the co-extraction of other compounds. Without this, the effects of the matrix can interfere in the actual response of the compounds of interest. One of the most used extraction techniques for the determination of pesticides in vegetable products is liquid–liquid extraction (LLE), followed by a "clean-up" with extraction in solid phase (SPE) or gel permeation chromatography (GPC). Recently, a general extraction procedure has been implemented, called "QuEChERS" (quick, easy, cheap, effective, rugged, and safe) due to its simplicity, the few stages of sample processing required, and its efficiency in the removal of impurities in complex samples [26,27].

## 2. Materials and Methods

### 2.1. Chemicals

The pesticides malathion 97.8 %, chlorpyrifos ethyl 99.1%, chlorothalonil 99.5%, and atrazine as the internal standard 99.2% were purchased from AccuStandar Inc. (New Haven, CT, USA). All solvents—toluene, acetonitrile, methanol, acetic acid, formic acid, and water—were obtained from Tedia (Carson City, CA, USA).

### 2.2. Calibration Graphs

Initially, there were individual solutions of each analyte at a concentration of 2000 µg/mL. From these, a standard solution of 20 µg/mL was prepared and further diluted until a stock solution of 20 ng/mL was obtained. The calibration samples were prepared by appropriately diluting the stock solution in the blank matrix. The concentrations of the calibration graphs ranged between 0.00011, 0.00032, 0.00107, 0.00218, 0.00641, and 0.01202 mg/kg.

To obtain the calibration graph for each compound, the method of least squares was used, considering the concentration ratio of each compound over the concentration of the internal standard versus the relationship of the areas of each standard over the area of the internal standard, which the regression equations were obtained for each compound. All correlation coefficients were greater than 0.99.

### 2.3. Sampling Site 

The study was carried out in the experimental field of the Faculty of Agricultural Sciences of the Autonomous University of the State of Morelos in the municipality of Cuernavaca (18.7317 N, -98.9182 O), Morelos, Mexico. The soil type is a vitric andosol soil (TV 14-2b). During the study period, the average temperature was 28 °C, the relative humidity was 65%, and there was illuminance of 10,752 lux.

### 2.4. Field Experiments

Before the experiment, a multi-residual pesticide analysis in soil and young cladodes was carried out with the purpose of determining the presence of residues. The Milpa Alta variety was used and planted on an area of 500 m^2^. The distance between plants was 40 cm and between rows 1.4 m. The agronomic management practices were those recommended for this crop [28].

### 2.5. Active Ingredients 

**Figure 1:**
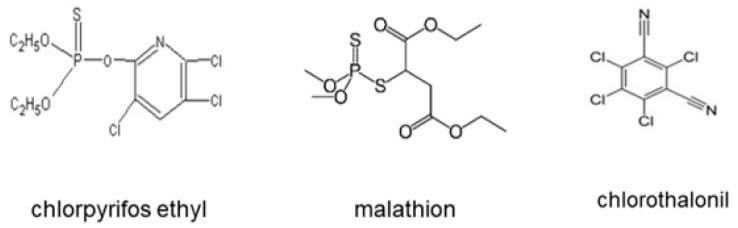
Structure of studied pesticides.

The commercial products used are showed in Table 1—two organophosphorus insecticide acaricides and chlorothalonil (contact fungicide with a broad action spectrum). All products are widely used in prickly pear pad production in Morelos, Mexico (Figure 1) [29,30].

Pesticide application was directed around the previously selected plants using a backpack sprayer with a constant pressure (40 psi) and a TXA8001VK Conejet^®^ VisiFlo^®^ hollow cone spray. Each treatment was applied three times with a one-week interval.

### 2.6. Experimental Design

A completely randomized block design was used with nine treatments and four repetitions for each treatment (Table 2). Each experimental unit had an area of 1.20 × 5 m, where six plants were selected from the center of each experimental unit for the collection of samples. 

The sample size was based on the Mexican prickly pear pad quality standard (NMX-FF-068-1998) [32] with a “B” size (21 to 24 cm), depending on the cladode length, for each repetition. During sampling step, a pad was taken from an untreated control. From treated plants, sampling was performed following the Codex Alimentarius guidelines [33]. A total of 1.5 kg of young cladodes from each plant was randomly taken by sampling the four sprayed plants (6 kg in total). The first sample was taken 1 h after pesticide application had dried and was labelled as 0 day. Afterward, cladodes were collected randomly at 3, 6, 10, and 15 days after application. Samples were placed in polyethylene bags and transported on ice to the laboratory. Each sample was cut into small pieces, then homogenized and frozen (−4 °C) in individual polyethylene bags until analysis.

### 2.7. Sample Preparation and Analysis

For the determination of pesticides in very complex matrices, the application of multi-waste methods was applied. These methods require rigorous analytical validation. Aware of this need, the General Directorate for Health and Consumers (SANTE) established the guidelines for the validation of an analytical method and the quality control procedures that must be carried out for the analysis of pesticide residues in agricultural products and their derivatives. The acceptance criteria for each of the validation parameters includes the repeatability of the method as a percentage of the coefficient of variation (RSD < 20%) and recovery percentages between 70–120% [34].

Samples were processed and analyzed according to a previously established methodology [33] and analyzed at the National Reference Center for Pesticides and Pollutants (CNRPC is its acronym in Spanish), which belongs to the National Service of Health, Safety, and Agrifood Quality (SENASICA is its acronym in Spanish). This is an accredited laboratory in the NMX-EC-17025-IMNC-2006/ISO/IEC17025:2005 standard that establishes the requirements for the testing and calibration practices laboratories must follow. 

The original young cladodes sampled were prepared as follows: 1.5 kg of cladodes from each plant was homogenized in a blender. After homogenization, 10 g was weighed in a 50 mL Polytetrafluoroethylene PTFE centrifuge tube, and 10 mL of acetonitrile containing atrazine (Internal Standard 0.000133 mg/kg), 1 g of sodium citrate (Na_3_C_6_H_5_O_7_), 1 g of sodium chloride (NaCl), and 4 g of magnesium sulfate (MgSO_4_), were added to each tube. Then each tube was vigorously stirred for 2 min (the QuEChERS method). 

From the previous solution, an aliquot of 3 mL was added to a plastic tube that contained 900 mg of magnesium sulfate (MgSO4), 150 mg of Primary Secondary Amine (PSA) (Thomas Scientific, NJ, USA), 150 mg of C18 resin (Sigma-Aldrich, St. Louis, MO, USA), and 80 mg activated carbon (St. Louis, MO, USA). It was then stirred for 1 min in a vortex and centrifuged at 3500 rpm for 2 min. The supernatant was filtered through a nylon membrane (0.2 µm), then filtrated prior to analysis by liquid chromatography.

### 2.8. Evaluation of Recovery Percentages

The recovery was determined using three replicates at one concentration level (0.003 mg/kg) for all compounds in the homogenized cladodes. Then, each sample was processed as explained in Section 2.7. With the concentration values observed after the extraction and the added concentration, recovery percentages were calculated for each compound. The results of the three replicates were used to calculate the accuracy of the method, expressed as relative standard deviation (% RSD).

### 2.9. Liquid Chromatography Analysis

A Waters ultra performance liquid chromatography-tandem mass spectrometer (UPLC-MS/MS; XEVO TQ-MS Mass Spectrometer, Waters Corporation, Milford, MA, USA) was used for the pesticide analysis. Pesticides were analyzed in an electrospray ionization in positive mode (ESI). Nitrogen was used as the desolvation gas at a flow of 100 L/h (500 ° C) and argon as the collision gas at a flow of 0.15 mL/min. A chromatographic column C18 (Acquity, UPLC BEH C18 1.7 µm, 2.1 × 100 mm, Waters Corporation, Milford, MA, USA) was used to separate the compounds. The column was kept at 60 °C, and the injection volume was 10 µL. Two eluents were used—0.1% formic acid in water-methanol (98:2) (A) and 0.1% formic acid in methanol (B). The flow rate was 0.35 mL/min. A linear gradient was used to elute the compounds: 0–2.30 min, 20% A: 80% B, 2.30–2.80 min, 100% B, 2.80–4.50 min, 20% A: 80% B. Collision cell energy and fragmentation voltage were optimized in the dynamic multiple reaction monitoring mode (MRM) for each pesticide (Table 3). To avoid false identification, it was important to check the retention time (RT), tolerance, and ion ratios obtained for each compound. The software Mass Lynx version 4 was used for instrument control and data acquisition. Each pesticide was tuned and the selected reaction monitoring (SRM) mode was used for quantification. The ratio of response was the ratio of the peak area of the SRMs. All the pesticide residues detected in the sampled cladodes were identified in accordance with the criteria specified by the European Commission SANTE (document 11945/2015)—RT (+/− 0.1 min) and ion ratios (< 30 %)—and compared against the reference [33].

### 2.10. Dissipation Curves

For each pesticide, two levels of concentration were applied: 0.240 and 0.407 mg/kg for malathion, 0.54 and 0.73 mg/kg for chlorpyrifos ethyl, and 0.46 and 0.87 mg/kg for chlorothalonil. Four samples of young cladodes were taken on the first day for each level and on days 3, 6, 10, and 15. 

The pesticide degradation kinetics was determined by plotting residue concentrations against time, and the maximum squares of correlation coefficients found were used to determine the equation of best-fit curves. For all samples, logarithmic relationships were found that corresponded to the first-order velocity equation. The persistence of pesticides is generally expressed in terms of half-life (t_1/2_), which is the time taken for the pesticide to reduce to 50% of its initial concentration. The rate equation was calculated from the first-order equation: C_t_ = C_0_e^−kt^, where C_t_ represents the concentration of the pesticide residues (mg/kg) at time (days), C_0_ represents initial concentration (mg/kg), and “k” is the first-order rate constant (per day) independent of C_t_ and C_0_. The half-life (t_1/2_) was determined from the “k” value for each experiment t_1/2_ = ln2/k. The data analysis was done with Excel office 2010.

## 3. Results and Discussion

### 3.1. Method Performance 

Method performance was evaluated by determining the percentages of recovery, precision, linear calibration, and limit of detection (LOD) according to EU guidelines. The recovery percentages for the studied pesticides were within the guidelines given in the “Guidance document on analytical quality control and method validation procedures for pesticide residues in food and feed” (SANTE/11945/2015) (70–120%). The LOD were between 0.00004 and 0.00008 mg/kg with RSD < 10%, which is congruent with established values [34] (Table 4).

### 3.2. Dissipation Curves

**Figure 2:**
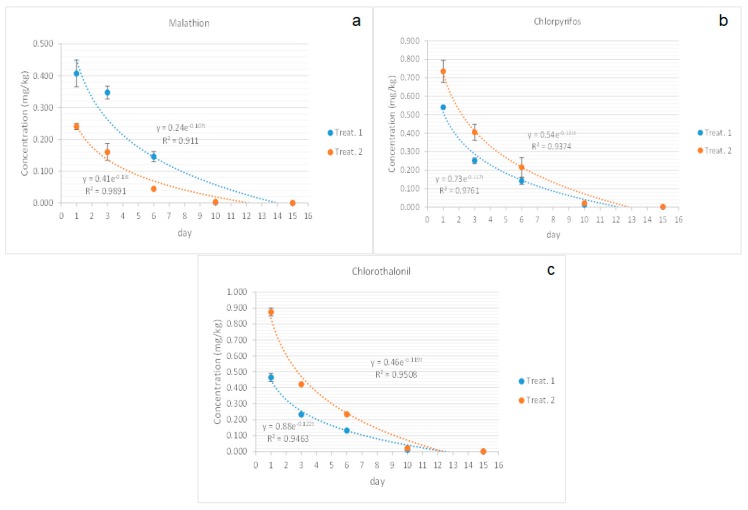
Dissipation curves for pesticides applied in two doses in nopal in Morelos, Mexico; (**a**) malathion, (**b**) chlorpyrifos, and (**c**) chlorothalonil.

The best-fitted model equations for the three pesticides in the sampled cladodes fit well to a first-order kinetic equation (Table 5). The pesticides dissipation curves are shown in Figure 2. 

#### 3.2.1. Malathion

The initial concentration level of malathion was 0.240 and 0.407 mg/kg for minimal and maximum dose, respectively. Both concentration levels for malathion were completely dissipated after ten days. The half-life was 5.77 and 5.33 days. The observed result for the dissipation rate (10 days) was similar to that reported in long bean, cauliflower, and eggplant samples, where the absence of malathion was reported after 12 days [35] (Figure 2a).

#### 3.2.2. Chlorpyrifos

For this compound, the sprayed initial concentration was 0.54 and 0.73 mg/kg for minimal and maximum dose, respectively. In tangerines (*Citrus reticulata* Blanco), the dissipation period was reported to be 65 days [36], and for grapes (*Vitis vinifera* L.) 15 days [37]. In prickly pear pads, the minimum dose was dissipated at ten days and for the maximum dose, at 15 days (Figure 2b). The half-life of chlorpyrifos in pepper plants was reported to be 2.64 days, for lettuce 3.92, for brassica chinensis 5.81, 3.0 for eggplant, and 5.45 for celery [38]. These differences in dissipation rates strongly depend upon the leaf characteristics of the vegetables evaluated, as well as environmental conditions [38]. 

#### 3.2.3. Chlorothalonil

The initial concentration was 0.46 and 0.87 mg/kg for minimal and maximum dose, respectively. The half-life was 5.8 days, which is congruent with previous reports in aerated soils and 5 to 15 days in flooded soils [39]. The period of dissipation for the minimum dose was 15 days and for the maximum was 20 days (Figure 2c). These results are similar to those reported on two tomato varieties grown in Poland with a 22 day period [40].

## 4. Conclusions

In Mexico, there are no records on the dissipation of the studied pesticides in prickly pear pads. For this reason, the results observed in this work enable suggestions to be made regarding the concentrations and conditions under which pesticides must be applied to protect consumers’ health.

In Mexico, no MRLs have been established for nopal, and for this reason, the current regulations suggest using a value of 0.01 mg/kg for unauthorized pesticides. Analyzing the results obtained under the established conditions, it was observed that in the four pesticides studied, the final concentrations were below the reference MRL, which suggests that these products can be safely applied in nopal production at the established concentrations.

According to the results observed in the dissipation curves, the harvest times for nopal crops should be after ten days when the minimum dose is used and after fifteen days when the maximum dose is used.

## Figures and Tables

**Table 1:** Classification and characteristics * of agrochemicals used in the prickly pear pad study.

Commercial Name	Active Ingredient	Action Mode	Chemical Group	Molecular Formula
Disparo^®^	Chlorpyrifos ethyl	Insecticide/acaricide	Organophosphate	C_7_H_7_CI_3_NO_3_PS C_22_H_19_CI_2_NO_3_
Malathion^®^ 1000	Malathion	Insecticide/acaricide	Organophosphate	C_10_H_19_O_6_PS_2_
Thalonil^®^ 75	Chlorothalonil	Fungicide	Chloronitrile	C_8_CI_4_N_2_

* Adapted from EURL-Data pool [31].

**Table 2:** Pesticide treatments, dose, in prickly pear pad crop.

Treatment	Active Ingredient	Dose
(*n* = 4, treatment)		mg/kg
T_11_	Malathion	0.240
T_2_	Malathion	0.407
T_3_	Chlorpyrifos ethyl	0.540
T_4_	Chlorpyrifos ethyl	0.730
T_5_	Chlorothalonil	0.460
T_6_	Chlorothalonil	0.870
T_7_	Untreated (negative) control	

**Table 3:** Optimized ultra performance liquid chromatography-tandem mass spectrometer (UPLC-MS/MS) parameters for the selected pesticides on prickly pear pad crop.

Analyte	RT (min)	First transition	Collision energy	Second transition	Collision energy	Quantifier ion
		(m/z)	(V)	(m/z)	(V)	
Malathion	6.3	173→99	12	173→127	5	173
Chlorpyrifos ethyl	7.1	314→286	20	314→258	10	324
Chlorothalonil	8.5	244.9→174.9	28	244.9→181.9	20	263

RT: retention time.

**Table 4:** Summary of the evaluated analytical parameters.

Analyte	LOD	% Recovery	% RSD
	mg/Kg	(*n* = 3)	(*n* = 3)
Chlorpyrifos ethyl	0.00004	84.5	9.54
Malathion	0.00006	87.8	6.54
Chlorothalonil	0.00005	86.5	7.35

LOD: limit of detection; RSD: relative standard deviation.

**Table 5:** Dissipation, t_1/2_, and correlation coefficients of studied pesticides.

Analyte	Minimal Dose			Maximum Dose		
	Kinetic Model ^a^	R^2^	t_1/2_	Kinetic Model ^a^	R^2^	t_1/2_
Malahion	C= 0.24e^−0.107t^	0.911	5.77	C = 0.41e^−0.13t^	0.9891	5.33
Chlorpyrifos	C= 0.54e^−0.131t^	0.9374	5.29	C = 0.73e^−0.117t^	0.9761	5.89
Chlotothalonil	C= 0.46e^−0.119t^	0.9508	5.8	C = 0.88e^−0.122t^	0.9463	5.68

^a^ Data was fitted to a first-order kinetic equation. R^2^: coefficient of determination; t_1/2_: half-life.
